# Image-Based Segregation of High-Quality Dragon Fruits Among Ripe Fruits

**DOI:** 10.3390/s26041113

**Published:** 2026-02-09

**Authors:** Coral Ortiz, Nikita Dapurkar, Vicente Alegre, Francisco Rovira-Más

**Affiliations:** 1 Rural and Agri-Food Engineering Department, Universitat Politècnica de València, Camino de Vera s/n, 46022 Valencia, Spain; frovira@dmta.upv.es; 2 Department of Horticulture, College of Agriculture, Marathwada Agriculture University, Parbhani 431402, India; 3 Greenvision, Catadau, 10 Benifayó, 46255 Catadau, Spain; greenvision@greenvision.es

**Keywords:** dragon fruit, image analysis, quality, firmness

## Abstract

The increasing demand for high-quality dragon fruit in the European market requires efficient quality assessment methods. This study explores a non-destructive image analysis approach for classifying ripe dragon fruits based on fruit ripeness and weight. A low-cost system equipped with visible and ultraviolet lighting was employed to capture images of two sets of samples of 60 and 92 ripe dragon fruits, extracting non-destructive parameters such as visible and ultraviolet perimeter, maximum and minimum diameter and area, and RGB color coordinates. Fruit destructive characterization parameters were also measured. The first set of samples was used to develop a discriminant classification model. In a first step, the main characterization magnitudes were confirmed. A ripening index was calculated based on soluble solid content and acidity. Then, a cluster analysis was used to segregate the fruits into three quality characteristics based on the ripening index and weight. In a second step, a step-by-step discriminant analysis was conducted to classify the fruits into the three quality categories (based on the laboratory-measured weight, soluble solid content and total acidity) using the non-destructive magnitudes extracted from the image analysis. The proposed classification system achieved an accuracy of nearly 85% of well-classified dragon fruits, effectively segregating dragon fruits into the three established categories. Furthermore, the established model could select the very high-quality dragon fruit (riper and larger fruits) with 93% of correctly identified products. A comparable procedure was subsequently applied to the additional set of samples (set 2), obtaining consistent results and confirming that image analysis magnitudes related to size and color enable fruit classification into the predefined weight- and ripeness-based categories. Compared to conventional destructive methods, this non-destructive approach offers a promising, cost-effective, and reliable solution for quality assessment. The findings highlight the potential for integrating smart technologies into fruit classification processes, during automatic harvest and postharvest operations, ultimately improving efficiency, reducing labor costs, and enhancing product consistency in the dragon fruit industry.

## 1. Introduction

Dragon fruit has recently been introduced to the European market. This herbaceous perennial climbing cactus, scientifically known as *Hylocereus* spp., is gaining attention from consumers [1]. This is not only due to its appealing red or pink color, economic significance, and rich content of antioxidants, vitamins, and minerals but also because of its cost-effective maintenance and high profitability. Recently introduced in the Mediterranean regions, the cultivation of dragon fruit has experienced rapid growth in Spanish Mediterranean areas [2]. Harvesting robots can mitigate labor shortages while offering a cost-effective solution to increasing labor expenses [3,4].

Image analysis is an important step in robotic fruit harvesting, allowing the autonomous system to identify and efficiently harvest fruits [5]. Fruit characteristics such as size, color, and shape are essential for autonomous harvesting [6]. Advanced computer vision techniques, including image and advanced data analysis techniques, allow robots to detect fruit presence, distinguish between different fruit types, and assess ripeness based on color, texture, and size [7]. Accurate image analysis helps minimize harvesting errors, such as picking unripe or damaged fruits, which can significantly impact crop yield and market value [8]. Furthermore, it enhances efficiency by optimizing robotic arm movements, ensuring precise fruit localization, and reducing time spent on unnecessary actions [9].

Machine learning models trained on large datasets improve fruit classification and defect detection, aiding in quality control during harvesting [10]. Additionally, image analysis assists in identifying occlusions caused by leaves or branches, allowing the robot to navigate complex environments without damaging surrounding vegetation [11]. By integrating multispectral imaging and depth perception, robots can evaluate fruit maturity beyond visual inspection, improving decision-making in real-time [12]. The implementation of high-resolution cameras, LiDAR, and thermal imaging further refine fruit detection accuracy, especially in varying lighting conditions [13,14]. Ultimately, image analysis is indispensable for the successful deployment of robotic fruit harvesting systems, leading to higher productivity, reduced labor dependency, and improved agricultural sustainability [15]. Robot fruit image analysis could be used to assess fruit quality before picking based on a decision system to harvest fruit with an optical ripeness stage [16]. Fruit size and color are the most commonly used criteria to distinguish between ripe and unripe produce [17].

Image analysis has proven successful in various fruit classifications. Previous studies achieved a 90.24% overall accuracy rate in categorizing persimmon fruit [18]. In mango classification, a combination of models like RF, LDA, SVM, and KNN, as well as an eight-layer convolutional neural network (CNN), achieved accuracy rates up to 95.67% [19]. In the case of mangosteen, a convolutional neural network model was proposed to detect defects and aid in sorting [20]. For classifying strawberries, ref. [21] used an RGB imaging system combined with convolutional neural networks and obtained accuracy higher than 90%. In reference to ultraviolet, ref. [22] proved that ultraviolet imaging can be used to identify hidden defects under mango peels, using geometric data extracted from the images to enable computer-based classification of apples between bruised and non-bruised.

An automatic dragon fruit quality classification system was proposed, utilizing image analysis, and demonstrated an impressive accuracy of 98.5%, surpassing manual classification six times [23], Other previous studies have also utilized a combination of image processing and artificial intelligence to recognize dragon fruit attributes, such as ripeness. Related to dragon fruit firmness, ref. [24] used non-destructive firmness parameters to identify two ripeness stages of dragon fruit related to color and other quality parameters. Previous authors defined algorithms based on shape, size, weight, color, and diseases to classify dragon fruits during sorting and grading operations, proposing three machine learning techniques (ANN, SVM, and CNN) [25]. A classifying system was also proposed by [26] using a model combining machine learning and image processing with convolutional neural networks, trained on a dataset of 1287 dragon fruit images. Considering that effective detection by machine learning models necessitates a substantial amount of data, ref. [27] gathered a dataset during four months from three distinct locations in Bangladesh, covering different stages from unripe to ripe and decayed fruits. However, in these previous studies, the classification performance was based solely on image datasets and was not validated against destructive laboratory analyses for fruit quality characterization. Despite previous advancements, image analysis has not yet been extensively explored for evaluating dragon fruit quality assessment in harvest and postharvest operations when most of the fruits are ripe. The objective of the present study was to address ripe dragon fruit quality classification using a low-cost image analysis system and identify the very high-quality fruits.

## 2. Materials and Methods

### 2.1. Vegetal Material

**Figure 1:**
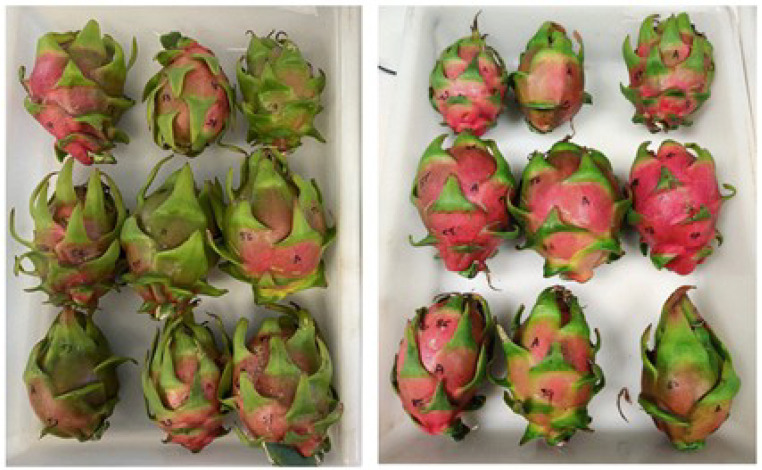
Example of fruits from two different days.

Two sets of samples of red dragon fruits (*Hylocereus costaricensis*) were tested, a first set of 60 fruits and a second set of 92 fruits. The fruits were collected from two local farms in Valencia (Spain) over two seasons. The fruits selected were free of physical damage, and uniform in appearance and size, and classified according to the ASEAN Standard for Dragon Fruit as Class I (grade B used in practical market grading systems to denote a standard/medium quality category) with red color for the skin and the flesh [28]. All samples corresponded to good-quality fruits, rather than extremes of the quality spectrum, as seen in Figure 1.

The fruits were all collected in a harvesting ripeness stage. The dragon fruits were transferred directly to the laboratory. After marking the fruits from 1 to 60, labeled on both sides, the fruits were stored in a cool chamber (6 °C, 85% RH) for 4 days. The fruits were examined using two distinct quality assessment methods, first with the non-destructive method and then with the destructive method. The fruits from the first set of samples were stored in a cold chamber and evaluated over a 7-day storage period at 2-day intervals. On each evaluation day, all samples underwent non-destructive measurements, and 15 fruits were additionally analyzed using destructive methods. The fruits from the second set of samples were harvested from the field when they reached the commercial ripeness stage. Three repetitions were carried out per sample.

### 2.2. Non-Destructive Assessment

**Figure 2:**
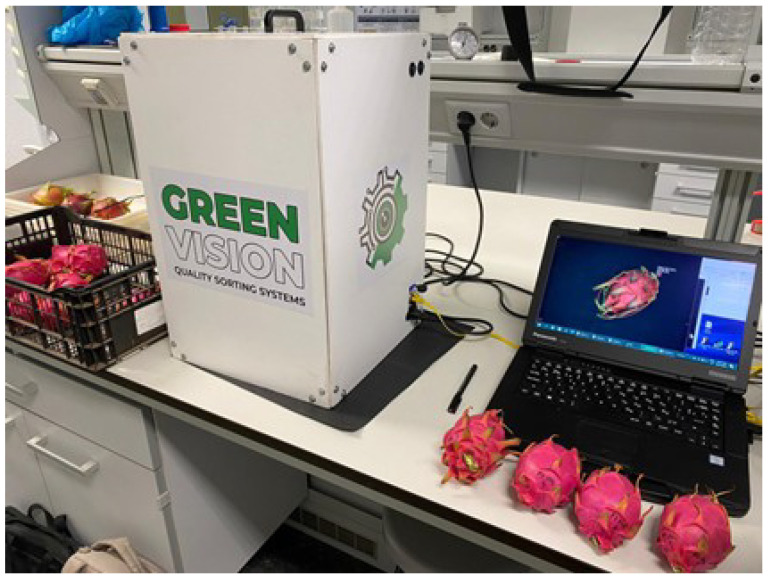
Imaging analysis system.

A low-cost non-destructive image analysis system with both visible and ultraviolet lighting (Greenvision, www.greenvision.com, accessed on 30 January 2026) was used, as shown in Figure 2.

The imaging system used in this study is an integrated laboratory platform combining multispectral hardware and dedicated control software. It includes a visible (VIS) camera (400–700 nm) capturing images under visible illumination and ultraviolet-induced fluorescence (UV). The camera is mounted on a movable structure, allowing reproducible framing and adjustment of working distance. The system employs multispectral illumination with visible LEDs and ultraviolet fluorescent tubes. Image acquisition is performed sequentially, with only one light source active at a time, preventing spectral interference and ensuring each image corresponds to a well-defined spectrum. The optical system uses 6 mm lenses to cover the sampling tray in a single capture while maintaining minimal distortion. Control software synchronizes the camera and illumination, manages automated acquisition sequences, and stores the images in a structured manner. The expressions RGB coordinates (Visible R coordinate, Visible G coordinate, and Visible B coordinate) refer to the average values of the red, green, and blue channels extracted from images captured in the visible spectrum. Similarly, the ultraviolet coordinates correspond to the mean RGB channel values obtained under UV illumination. Mean RGB coordinates were computed from the segmented fruit region as the average pixel intensity of each channel. In VIS mode, these values correspond to the chromatic content captured directly by the camera, whereas in the UV mode, they quantify the visible fluorescence induced by ultraviolet excitation. All RGB values were extracted exclusively from the object mask to avoid background influence. Fruit segmentation in VIS and UV images was carried out using a YOLO-based deep learning model implemented in PyTorch 2.7.0 trained to detect the fruit silhouette. The network operates in inference mode to generate binary masks, ensuring robustness to illumination variability, background independence, and cross-spectral consistency. These masks served as the region of interest for all chromatic and geometric analyses. The processing pipeline consisted of image acquisition, YOLO segmentation, mask generation, and feature extraction.

**Figure 3:**
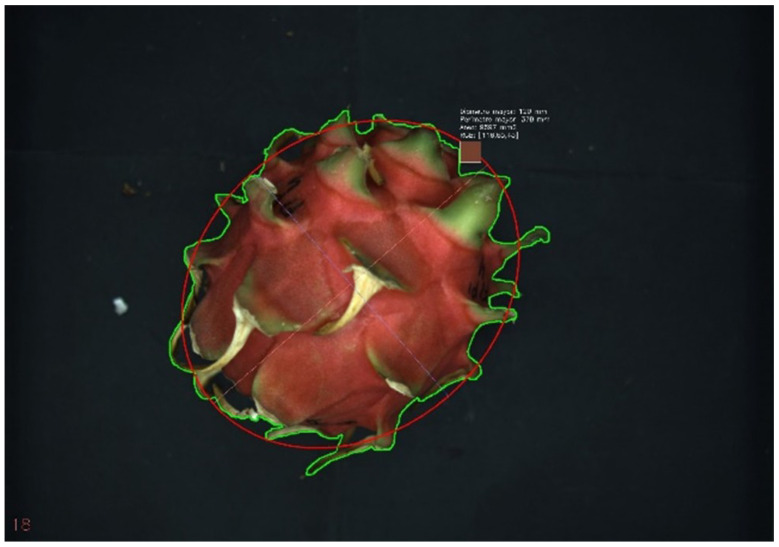
Dragon fruit image obtained from Greenvision imaging system with physical parameters.

Visible aSnd UV images of dragon fruit on each alternate day of storage were taken by placing dragon fruit directly into the image chamber. Two images were captured from each side of the fruit and each type of light. The image analysis parameters obtained were visible and ultraviolet perimeter, maximum and minimum diameter, and color coordinates RGB (Figure 3).

After the non-destructive image analysis was finished, weight loss was measured using a digital balance (Mettler Toledo AL104 electronic balance, Im Langacher 44, Horticulturae 2023, 9, 1286 5 of 11 8606 Greifensee, Switzerland).

### 2.3. Destructive Quality Assessment

Laboratory reference destructive variables were measured to assess dragon fruit quality using standardized magnitudes. Destructive firmness was measured using a universal stress–strain machine (Ibertest, model IBTH 2730, www.ibertest.es), with a constant speed of 1.67 m $s^{-1}$. The firmness of each fruit was measured at three different points of the equatorial area using a 0.008 m probe. Three repetitions were carried out per fruit. Using a blender, each dragon fruit was juiced separately, and the filtered liquid juice was utilized to calculate the soluble solid content. The digital refractometer was utilized to measure the soluble solid content (Atago model PAL-3; Atago Co., Tokyo, Japan). Results are expressed in °Brix, which indicates the concentration of total soluble solids in the fruit juice, primarily sugars but also other soluble compounds. Tritatable acidity was measured with an automatic titrator (Mettler Toledo AL104 electronic balance, Im Langacher 44, 8606 Greifensee, Switzerland). A known volume of fruit juice was titrated with a standardized sodium hydroxide (NaOH) solution until reaching an endpoint of pH 8.1. The results were expressed as grams of citric acid equivalent per 100 g of sample.

### 2.4. Data Analysis and Modeling

The statistical analysis was performed by using a commercially available software package (Statgraphics Centurion 18 Software, version 18.1.13 (Statgraphics Technologies, Inc., The Plains, VA, USA)). One-way analysis of variance was used to assess the effect of the treatments on the destructive measured variables. A step-by-step discriminant analysis based on the non-destructive variables (visible and ultraviolet perimeter, maximum and minimum diameter, and area, and color coordinates) extracted from the image analysis system was used for the classification of the fruits into different ripeness categories according to the testing days.

## 3. Results

In Table 1 and Table 2 the measured variables (average values and standard deviation) are shown, from the destructive and non-destructive characterization analysis and from the image analysis, from sample sets 1 and 2.

**Figure 4:**
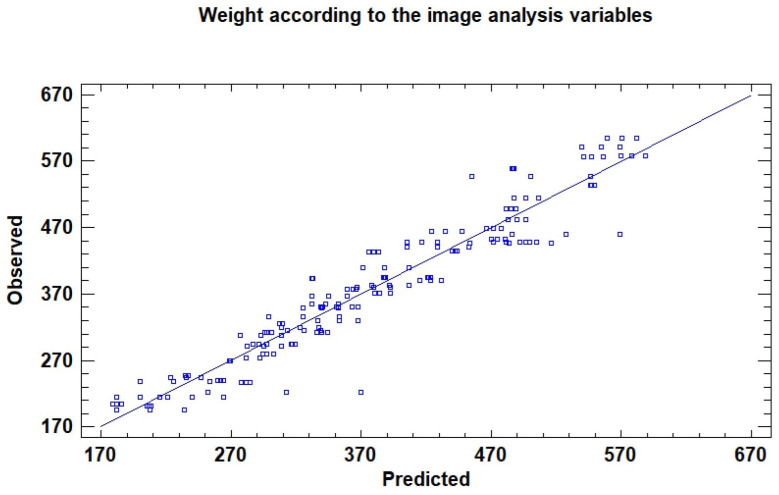
Observed-versus-predicted values from the PLS model predicting weight according to the visible and ultraviolet variables obtained from the image analysis, five latent components, R square of 90.5%.

One of the main parameters used to evaluate dragon fruit quality is weight. In a first step, this variable was analyzed through a partial least square (PLS) model established to determine fruit weight from the image analysis variables (Table 3 and Figure 4). Five latent components were extracted, and model stability was assessed through leave-one-out cross-validation. The first component explained 43.71% of the variance in X and already captured 81.19% of the variance in Y, showing that most weight variability is associated with the main image features. The subsequent components progressively improved the model, raising the cumulative R^2^ to 91.53%, while the predictive R-squared obtained from PRESS reached 90.46%. The consistent reduction in MSE and PRESS across components confirms the robustness of the model and its ability to capture the relationship between image-derived features and fruit weight. Additionally, the observed-versus-predicted plot illustrates the strong agreement between measured and estimated values, with the five-component PLS model achieving a predictive R-squared of 90.5%, demonstrating high accuracy in estimating fruit weight from the visible and ultraviolet image variables. Overall, these findings confirm that weight, a key quality parameter, can be reliably estimated from image analysis features, supporting the feasibility of non-destructive prediction.

The quality variables measured by destructive test were analyzed. An analysis of variance was developed to study the effect of the factor testing day on the destructive variables. Significant differences were found in the soluble solid content and the total acidity according to the testing day. During the storage period, as expected, sugar content increased, and total acidity decreased. However, no significant differences were found in the mechanical firmness, showing a wide variability between fruits. Following previous studies [29,30], a ripening index was established based on the destructive variables (soluble solid content and total acidity), according to Equation (1).

(1) $$\mathrm{Ripening}\;\mathrm{index}=\frac{\mathrm{Soluble}\;\mathrm{solid}\;\mathrm{content}\;\left({}^{\circ}\mathrm{Brix}\right)}{\mathrm{Total}\;\mathrm{acidity}\;\left(g/L\right)\times 100}$$

**Figure 5:**
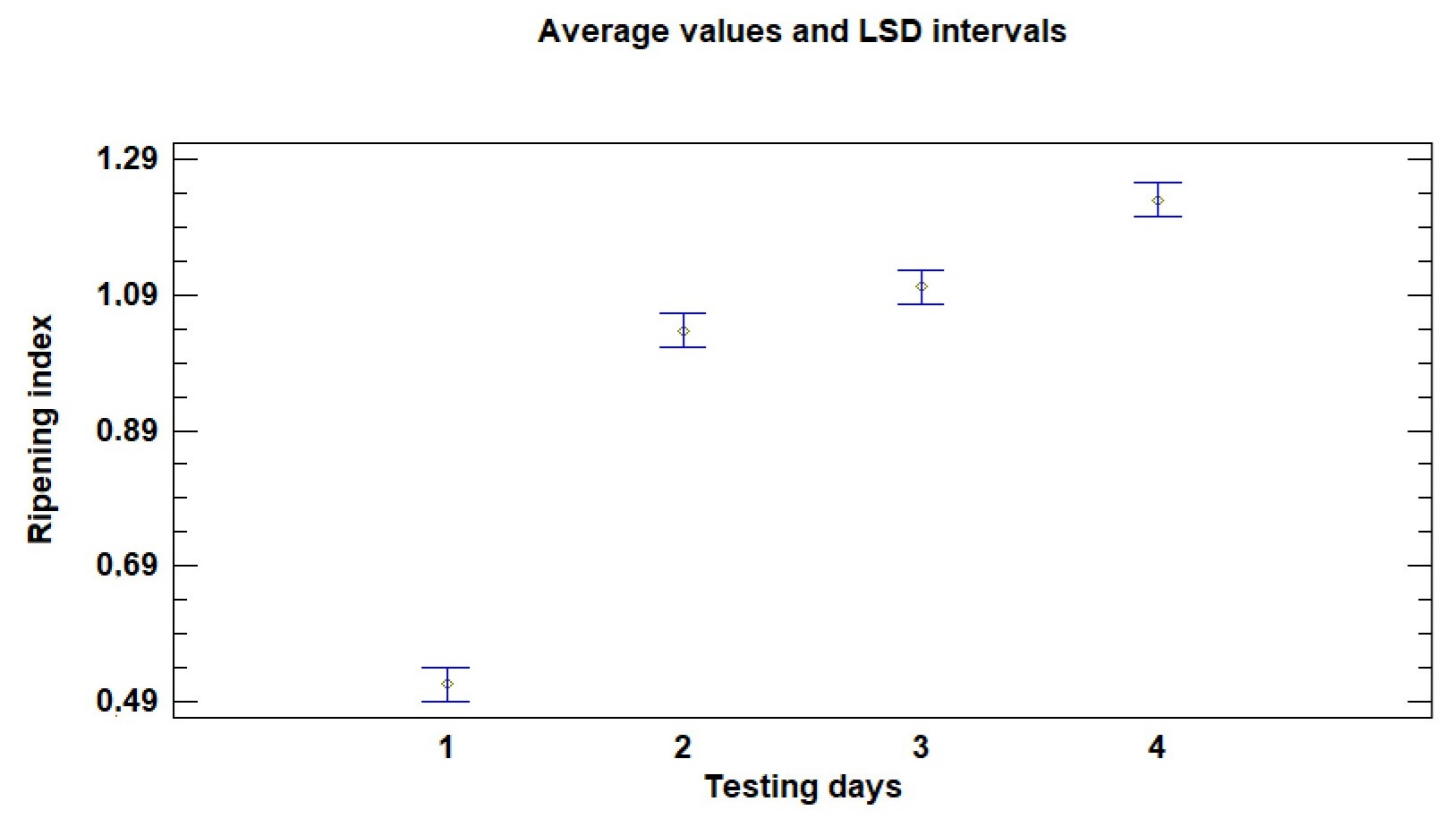
Influence of testing day on the ripening index (average values and LSD intervals). Lowercase letters denote significant differences among the different harvesting times, as determined by a Duncan test (*p* < 0.05).

During the storage period, significant differences were found in the ripeness index according to the testing day, as shown in Figure 5.

The same procedure was applied to the two sets of samples. First, the quality categories for each season were defined using a cluster analysis based on laboratory-measured fruit weight and ripening index, establishing the season-specific categories. Then, a discriminant analysis was performed using the non-destructive variables obtained from image analysis to classify the fruits into the previously defined categories. Cluster analysis based on laboratory-measured fruit weight and ripening index (related to soluble solids and total acidity) identified three distinct categories of dragon fruit quality using Ward’s method and squared Euclidean distance after standardization. Category 1: “Unripe” included fruits with a very low ripening index, indicating low sugar content and poor flavor. Category 2: “Large-sized ripe” comprised fruits with an acceptable ripening index and high weight, corresponding to fruits of larger size with good flavor. Category 3: “Small-sized ripe” contained fruits with an acceptable ripening index but low weight, corresponding to fruits of smaller size with good flavor. Operationally, samples were assigned to the category whose centroid (mean ripening index and weight in standardized space) was closest, following the Ward clustering procedure. Fruits with a ripening index of about 0.8 or lower are classified as “Unripe.” Among fruits with a ripening index around 0.8 or higher, those weighing less than approximately 400 g are categorized as “Small-sized ripe,” while those weighing about 400 g or more are categorized as “Large-sized ripe”.

**Figure 6:**
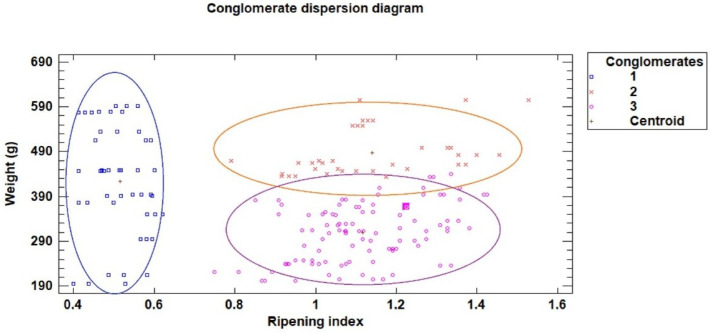
Conglomerate dispersion diagram, according to weight and ripeness index.

**Figure 7:**
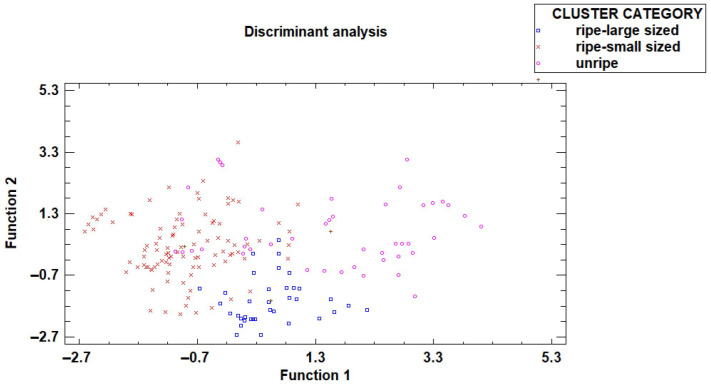
Discriminant analysis to segregate into the three categories based on the non-destructive variables obtained from the image analysis.

Based on the three groups determined using a cluster analysis technique (Ward method and squared Euclidean distance), as seen in Figure 6. The category number 1 (shown in blue in Figure 6) had a very low ripening index (low sugar content and high acidity), and was labeled as “unripe”. The category number 3 (shown in purple in Figure 6) had an acceptable ripening index but low weight, and was labeled as “small sized-ripe”. And the category number 2 (shown in orange in Figure 6) had an acceptable ripening index and high weight, and was labeled as “large sized-ripe”. In a second step, a step-by-step discriminant analysis was undertaken, utilizing the non-destructive variables obtained by image analysis (visible and ultraviolet perimeter, maximum and minimum diameter, and color-coordinated RGB) to classify the fruit into the three categories established based on ripening index and weight. A percentage of 70% of the data was used for building the model and 30% for validating the model. The results showed that 84.40% of the fruits were accurately classified into their respective categories by the model (Table 4 and Figure 7). Additionally, 74.10% of the fruits from the validating set were correctly classified using the developed model. This shows the discriminant analysis effectively uses non-destructive factors to correctly classify dragon fruits according to their quality based. The results showed that more than 90% of the fruits from “ripe-large sized” were correctly classified, more than 85% from the “ripe-small sized” and nearly 80% of the “unripe” dragon fruits. The variables selected in the step-by-step analysis to classify the fruits into the quality categories were the ultraviolet extracted variable perimeter, area, and minor diagonal and the visible color coordinate G.

The same procedure was carried out with the additional set of samples. In a first step, the fruits were classified according to the three categories previously defined according to weight and ripeness index. In a second step, a step-by-step discriminant analysis was developed to classify the fruits into the quality categories based on the image analysis parameters, as shown in Table 5. The variables’ visible perimeter, visible area, visible minor diagonal and Coordinate R were selected obtaining a 79.71% of well-classified fruits, indicating that non-destructive image analysis parameters related to size and to color R/G could define the quality category of commercial dragon fruits.

## 4. Discussion

Compared to conventional sensory evaluation methods, mechanical and chemical destructive tests have been proposed as alternative methods for assessing fruit quality attributes. Chemical analysis utilizing destructive laboratory devices is a recognized technique for evaluating the quality attribute of fresh fruits, especially in dragon fruit among other fruits. Determining the soluble solid content of fruit juice is a commonly employed approach to evaluating fruit juice quality, particularly about taste attributes. Soluble solid content indicates the sugar concentration in the juice, which is closely associated with sweetness and overall flavor perception. These chemical tests, while providing valuable insights into fruit quality attributes, typically require the destruction of the fruit sample. Where in-depth analysis is necessary, mechanical and chemical destructive tests remain indispensable tools for fruit quality assessment.

A non-destructive low-cost system that efficiently evaluated quality of dragon fruit has been proposed. This innovative approach aims to provide accurate and efficient quality evaluation without causing damage to the fruit. The validation process of this novel non-destructive device involves segregating fruits into quality categories. This validation is achieved by analyzing data collected from visible and ultraviolet images. In the discriminant analysis, the objective was to identify which image-derived variables best distinguish among the three previously established fruit quality categories based on the destructive variables. A stepwise selection procedure was applied to 16 independent variables derived from visible and UV images. The analysis revealed that the variables related to size and to R/G color were significant predictors. The size variables identified by the model (visible and UV perimeter, area, and minor diagonal) describe external fruit size and geometry. These features are determined by fruit growth processes, mainly cell division and cell expansion, which define the final dimensions of the fruit. Besides, the green and red color coordinates represent surface color information linked to chlorophyll presence, directly related to fruit ripeness stage. The results indicated that this non-destructive system could achieve classification accuracies of 84.40% and 79.71% (from the first and second sets of samples, respectively) classifying fruits into three quality categories (“unripe,” “ripe-small sized,” and “ripe-large sized”, previously established by cluster analysis-based ripening index and weight) (Figure 7) based on the image analysis parameter. AAdditionally, 92.30% and 95.12% (from the first and second sets of samples, respectively) of the high-quality dragon fruits were correctly identified. This fact is crucial considering the importance of not misclassifying these products that could be sold for a higher added value. Furthermore, the identification of these high-quality dragon fruits, produced in the Mediterranean region, could help encourage consumer loyalty in the purchase of these products. Comparing the obtained results with previous reported studies, higher accuracy levels in classifying dragon fruits could be found. However, these studies included ripe and also very unripe fruits, as [26] found 96.38% of well-classified fruits in three categories, including clearly unripe fruit. In the presented research, the fruit classified as “unripe” were already in a harvest ripeness stage. Classification methods play a pivotal role in fruit quality assurance protocols, contributing significantly to the precision and reliability of assessments. The ability to accurately segregate fruits into different quality categories using non-destructive methods enhances efficiency in harvest and postharvest handling processes, especially for high-added-value tropical fruits produced in Europe and destined for the European market.

This study presents certain limitations that should be considered when interpreting the results. First, the analysis was conducted on a single variety of dragon fruit, which may restrict the applicability of the findings to other varieties with different morphological or color characteristics. Future research should include a broader range of varieties to validate the robustness of the proposed approach. Second, image acquisition was performed under controlled lighting conditions to ensure consistency in color and texture analysis. While this improves accuracy, it may limit the generalization of the method to field environments where lighting is variable. Further work should explore adaptive image processing techniques to address these challenges. Despite these limitations, the proposed methodology has significant potential for practical applications. In particular, the integration of image analysis into robotic harvesting systems could enhance fruit detection and quality assessment, contributing to greater automation and efficiency in precision agriculture.

## 5. Conclusions

The use of tools to assist fruit production is required to ensure good-quality fruit. The present study introduces a non-destructive classification system for ripe dragon fruit using a low-cost system. Utilizing the data obtained from image analysis, the classification process employed step-by-step discriminant analysis. This analytical approach was capable to classify ripe dragon fruits into three quality categories (previously established by cluster analysis-based ripening index and weight) with an accuracy of 84.40% and 79.71% for well-classified fruits, based on the variables extracted from the image analysis related to fruit size and geometry, and color. Furthermore, the established model could select the very high-quality dragon fruit (riper and larger fruits) with 92.30% and 95.12% of correctly identified products. In conclusion, the proposed non-destructive dragon fruit classification system, utilizing image-based parameters, presents a promising solution to accurately categorizing ripe dragon fruit based on their visual attributes, offering both cost-effectiveness and reliability in fruit classification processes, for automatic harvest and postharvest operations. This advancement has the potential to streamline quality control processes in the dragon fruit industry, leading to improved efficiency and product consistency. Continued research and refinement of this system could further improve its performance and applicability in commercial settings.

## Figures and Tables

**Table 1:** Measured variables (average values and standard deviation) from destructive analysis, non-destructive characterization, and image analysis of samples from set 1.

Measured Variable	Average	Std. Deviation
Destructive firmness (N/mm)	3.5	0.6
Soluble solid content (°Brix)	12.9	1.6
Total acidity (g/L)	0.1	0.1
Weight (g)	373.6	109.4
Area visible	9471.1	2112.7
Perimeter visible	646.2	163.5
Major diagonal visible	134.2	14.8
Minor diagonal visible	87.8	10.8
Coordinate R visible	56.1	12.4
Coordinate G visible	59.8	6.4
Coordinate B visible	31.0	3.6
Area ultraviolet	9917.7	2161.0
Perimeter ultraviolet	594.8	149.9
Major diagonal ultraviolet	138.3	15.3
Minor diagonal ultraviolet	91.1	10.9
Coordinate R ultraviolet	22.8	7.8
Coordinate G ultraviolet	74.2	26.0
Coordinate B ultraviolet	65.4	22.4

**Table 2:** Measured variables (average values and standard deviation) from destructive analysis, non-destructive characterization, and image analysis of samples from the additional set of samples, set 2.

Measured Variable	Average	Std. Deviation
Destructive firmness (N/mm)	3.3	0.5
Soluble solid content (°Brix)	13.0	1.7
Total acidity (g/L)	0.1	0.1
Weight (g)	295.6	82.9
Area visible	7994.4	1630.4
Perimeter visible	625.3	127.3
Major diagonal visible	112.5	12.6
Minor diagonal visible	95.3	9.8
Coordinate R visible	56.3	6.3
Coordinate G visible	48.7	7.7
Coordinate B visible	35.5	5.4

**Table 3:** Summary of the components with percentage of variance explained in X and Y, R-squared, mean squared error (MSE), and prediction error (PRESS).

Component	% Variance in X	% Variance in Y	R^2^	MSE	PRESS
1	43.71	81.19	81.19	2324.4	80.48
2	27.60	2.96	84.16	1985.55	83.32
3	10.14	4.31	88.46	1503.93	87.37
4	5.88	2.42	90.88	1210.39	89.83
5	5.40	0.65	91.53	1135.10	90.46

**Table 4:** Classification matrix according to the three established fruit quality categories based on the image analysis variables, set of samples 1.

Actual Category	Ripe—Large-Sized	Ripe—Small-Sized	Unripe
Ripe—Large-Sized (36)	93.31% (34)	3.85% (1)	3.85% (1)
Ripe—Small-Sized (98)	8.20% (8)	85.25% (84)	6.56% (6)
Unripe (45)	5.26% (2)	15.79% (7)	78.95% (36)

**Table 5:** Classification matrix according to the three established fruit quality categories based on the image analysis variables, additional set of samples 2.

Actual Category	Ripe-Large Sized	Ripe-Small Sized	Unripe
Ripe-Large Sized (123)	95.12% (117)	0.00% (0)	4.88% (6)
Ripe-Small Sized (33)	9.09% (3)	87.88% (29)	3.03% (1)
Unripe (120)	14.17% (17)	0.83% (1)	85.00% (102)

## Data Availability

The raw data supporting the conclusions of this article will be made available by the authors on request.
